# The Effects of Different Grafting Periods, Method, and Environmental Factors on the Grafting Propagation of *Carpinus betulus*

**DOI:** 10.3390/plants15040604

**Published:** 2026-02-13

**Authors:** Yuanlan Zhang, Weixu Meng, Jiaxin Ji, Kun Wang, Cheng Zhang, Zunling Zhu, Qianqian Sheng

**Affiliations:** 1 College of Life Sciences, Nanjing Forestry University, Nanjing 210037, China; nlzyl2020@163.com; 2 Co-Innovation Center of Sustainable Forestry in Southern China, Nanjing Forestry University, Nanjing 210037, China; xiaomaizi666@njfu.edu.cn (W.M.); 788jijiaxin144311@njfu.edu.cn (J.J.); zhangcheng@njfu.edu.cn (C.Z.); zhuzunling@njfu.edu.cn (Z.Z.); 3 College of Landscape Architecture, Nanjing Forestry University, Nanjing 210037, China; 4 Jinpu Research Institute, Nanjing Forestry University, Nanjing 210037, China; 5 Digital Innovation Design Research Center, Nanjing Forestry University, Nanjing 210037, China; 6 College of Art & Design, Nanjing Forestry University, Nanjing 210037, China

**Keywords:** *Carpinus betulus*, grafting method and period, light and humidity, graft survival rate

## Abstract

*Carpinus betulus* is an important ornamental landscape tree species with colorful foliage. It is widely used in landscaping due to its upright tree shape, significant seasonal changes, and good tolerance to pruning. Propagation methods for *C. betulus* include grafting, cutting, and seeding. However, the germination rate of seeding is low, and the rooting of cuttings is difficult; moreover, plant tissue culture techniques are complex, and the key technologies have not been disclosed. Grafting has therefore become the primary means of propagation. However, enabling the rapid reproduction of *C. betulus* through appropriate grafting methods and in appropriate environments remains an urgent issue to be addressed. In this study, *Carpinus turczaninowii* was used as a rootstock to graft *C. betulus*, and the effects of the grafting periods, technique, and environmental conditions on the survival rate of grafted *C. betulus* were discussed. The results showed that branch grafting (cleft graft and whip-and-tongue graft) performed in March to April and August to November resulted in the highest survival rates, whereas budding grafts (chip budding and patch budding) were more suitable in May and June. Increasing ambient humidity was a key measure for improving graft survival rates and germination rates. In terms of grafting survival rate, germination rate, and leaf growth, humidification and treatment with 60–70% light transmission had better results than treatment with natural humidity or 20–30% light transmission and full light treatment under humidification conditions. Under low-light conditions, increasing air humidity had a particularly pronounced effect on promoting the growth of grafted seedling branches. In the future, further research should be conducted on the molecular mechanism mediated by soil environment and temperature changes for the successful grafting of *C. betulus*, providing a theoretical basis for the propagation and cultivation of *C. betulus*.

## 1. Introduction

*C. betulus* belongs to the genus Carpinus of the Betulaceae family. It is a deciduous tree with a full crown and dense foliage, golden-yellow autumn leaves, and yellowish-brown fruit clusters [1]. This species is widely cultivated across temperate regions. Owing to its elegant shape, varying from that of a tower to an oval crown, and rich seasonal changes, *C. betulus* is regarded as one of the most important landscape trees [2], often used as a street tree, shade tree, hedge, or bonsai [3].

In the asexual reproduction of *C. betulus*, grafting is the preferred technique. Although *C. betulus* can be propagated via seeds, the resulting seedlings exhibit high genetic variability, leading to inconsistent crown shapes and a loss of specific ornamental traits (e.g., fastigiate form or foliage color) [4]. Therefore, clonal propagation is essential to maintain true-to-type cultivars. However, since cutting propagation has proven difficult due to the poor rooting ability of this species, grafting has become the most effective solution. This process promotes healing and survival by attaching the buds or branches of a superior cultivar (the “scion”) to a plant with a robust root system (the “rootstock”) [5]. This asexual reproduction method can not only rapidly produce high-quality plants but also improve plant stress resistance, pest and disease resistance, and ornamental value [6]. Consequently, grafting is widely applied in the propagation of forest trees, fruit trees, and ornamental plants [7]. By selecting appropriate rootstocks, grafting can also regulate tree vigor (for example, dwarfing or greening) to meet diverse cultivation needs [8]. In recent years, due to advances in temperature and humidity control, along with light regulation technologies, graft propagation has become a key modern nursery technique, especially for expanding plants that are difficult to propagate sexually or have long generation cycles.

In practice, grafting success depends primarily on the affinity between the rootstock and scion, which is characterized by the rapid formation of vascular connections [9,10]. Furthermore, this process is significantly influenced by the grafting timing, technique, and environmental conditions, including temperature, humidity, and light [11]. Grafting techniques are generally categorized into branch grafting and bud grafting. Common branch graft methods include cleft graft and whip-and-tongue graft, while bud grafting methods mainly include chip budding and patch budding [12]. While branch grafting can theoretically be performed throughout the year, it is predominantly conducted in spring and autumn for commercial seedling production. For deciduous species, early spring is the optimal period for branch grafting as it coincides with bud swelling and the reactivation of the vascular cambium. Winter branch grafting is usually performed indoors, and the grafted seedlings are transplanted outdoors in spring. Bud grafting is the preferred method for achieving seedlings in the same year in production; its survival rate is closely related to the degree of “peeling” and the activity of the cambium layer. It can be carried out in spring, summer and autumn. As the epidermis is prone to peeling during the growth period, bud grafting is often chosen in summer.

During the grafting process, environmental factors are critical variables that regulate physiological healing between the scion and rootstock, thereby directly dictating the quality of callus formation and the graft survival rate [13]. Specifically, temperature, humidity, and light interact to form a complex physiological and ecological regulatory network [11]. Among these, temperature is the most influential external factor affecting callus induction and development. Although the optimal temperature range varies slightly among species, callus proliferation is generally most vigorous at approximately 25 °C. In contrast, excessive heat tends to inhibit growth, whereas relatively lower temperatures favor callus formation [14]. For instance, Dani reported that prolonged exposure to high temperatures resulted in a significant decrease in callus biomass in cotton [15].

Humidity is another critical environmental factor that maintains water balance and cellular activity within the graft [16]. Desiccation of the wound interface leads to cell necrosis and suppresses callus induction, whereas water loss in the rootstock compromises its vigor and growth [17]. Nie et al. found that appropriate moisture-retaining measures, such as coating the graft interface with mud, wrapping with wet sawdust, wax sealing, or tying with plastic film, effectively increased the local humidity around the graft union and promoted healing [18]. Existing research indicated that there were significant differences in the optimal range of environmental humidity for different tree species during the grafting stage. For instance, in the experiment conducted by Liu Zhenliang to explore the factors influencing the survival rate of *Pistacia vera* grafting and the growth conditions of the grafted trees, it was found that the survival rate of *P. vera* was the highest under a relative humidity of 60–70% [19], whereas in her research, Zhang Naiyan found that *Camellia petelotii* grafting required 85–90% humidity, confirming that the optimal humidity had definite species specificity [20].

Meanwhile, light is a key environmental factor regulating the healing of grafts, which can determine the formation and quality of callus by influencing the metabolic activity of plant tissues and cell division [21]. Appropriate light exposure helped promote nutrient supply and signal transduction in scions and rootstocks, promoting callus differentiation and accelerating the reconnection and regeneration of vascular bundles [22,23]. Moreover, relatively high light intensity can upregulate proteins related to ion binding, amino acid metabolism, and defense responses, thereby strengthening vascular bundle reconnection and improving the survival rate of grafting [24].

The propagation methods of *C. betulus* include grafting, cuttings and seeding, etc. However, seed germination rates are naturally low, cuttings are recalcitrant to rooting, and tissue culture remains technically complex due to the lack of established protocols. Consequently, grafting has become the primary propagation approach for this species. However, the specific physiological constraints imposed by seasonal changes and microclimatic interactions on graft healing remain to be elucidated. Therefore, we hypothesized the following: (1) Graft compatibility in *C. betulus* is strictly delineated by the seasonal activity of the vascular cambium, making the dormant phases (spring/autumn) optimal for branch grafting and the active growth phases (early summer) suitable for bud grafting, while mid-summer high temperatures impose a thermal barrier to union formation. (2) Environmental humidification exerts a synergistic effect with moderate light intensity by minimizing transpirational water loss to maintain the scion’s water balance, thereby optimizing the energy conditions required for vascular regeneration. To test these hypotheses, we utilized *C. turczaninowii* as rootstocks to systematically evaluate the effects of grafting period, method, and environmental regulation on survival and growth, aiming to provide a mechanistic basis for the efficient large-scale propagation of this species.

## 2. Results

### 2.1. Effects of Humidity and Light Treatment on Survival Rate and Germination Rate of Cut-Grafted Seedlings of C. betulus

**Figure 1:**
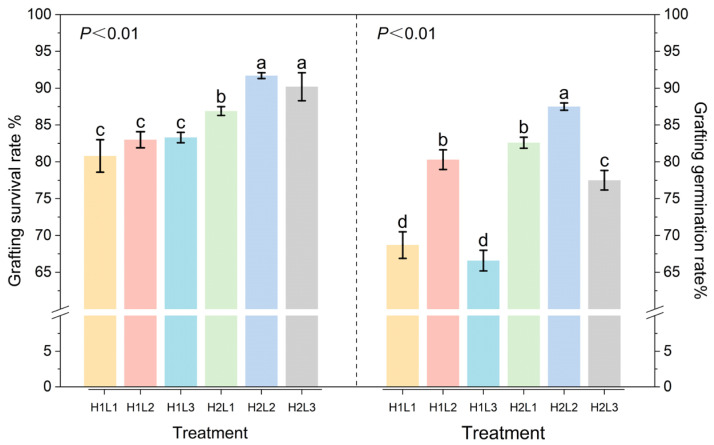
Effects of the survival rate and germination rate of *C. betulus* grafted under different combinations of humidity and light. Note: H1L1 (CK) is the “natural humidity + full light” treatment; H1L2 is the “natural humidity +60–70% light transmission” treatment; H1L3 is the “natural humidity +20–30% light transmission” treatment; H2L1 is the “humidity increase + full light” treatment; H2L2 is the “humidity increase +60–70% light transmission” treatment; H2L3 is the “humidity increase +20–30% light transmission” treatment; each value is the mean ± SE (*n* = 3). Different lowercase letters above the bars indicate significant differences between treatments (*P* < 0.05) according to Duncan’s multiple range test. Treatments sharing the same letter are not significantly different. Treatments sharing the same letter are not significantly different (e.g., ‘a’ is significantly different from ‘b’).

Different treatments had extremely significant effects on the survival rate and germination rate of *C. betulus* grafting (*p* < 0.01) (Figure 1). Under the same lighting conditions, the germination rate and survival rate of the grafted seedlings of *C. betulus* treated with H2 were significantly higher than those treated with H1. In terms of grafting survival rate, the average survival rate of 6 treatments exceeded 80%, with H2L2 and H2L3 treatments both exceeding 90%, significantly higher than the other treatments, although the difference between the two was not significant. Under H1 treatment, the survival rates of grafted seedlings under 3 light treatments did not differ significantly. In terms of grafting germination rate, H2L2 treatment had the highest average germination rate, at 87.5%. Under the same humidity conditions, L2 treatment had a significantly higher germination rate than L1 and L3 treatments.

### 2.2. Effects of Humidity and Light Treatment on Branch Length and Diameter Growth of C. betulus Grafting Seedlings

Two-way ANOVA results showed that treatment days (T) and different treatments (TM) had significant effects on branch length and diameter growth of *C. betulus* grafted seedlings (*p* < 0.01). The interaction (T × TM) had a significant effect on branch length (*p* < 0.01), but no significant effect on shoot diameter (*p* = 0.88).

**Figure 2:**
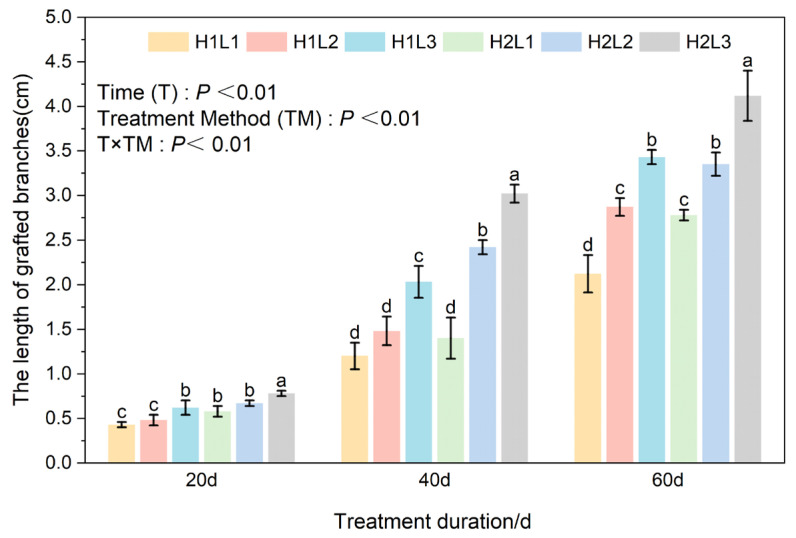
Effects of different light and humidity combinations on branch length of grafted seedlings of *C. betulus*. Different lowercase letters above the bars indicate significant differences between treatments (*P* < 0.05) according to Duncan’s multiple range test. Treatments sharing the same letter are not significantly different. Treatments sharing the same letter are not significantly different (e.g., ‘a’ is significantly different from ‘b’).

In terms of branch length (Figure 2), during the entire treatment period H2L3 treatment showed significantly higher branch length than other treatment groups, reaching 4.12 cm at 60 d. Under the same light treatment, except for 40 d of H1L1 and H2L1, branch length under H2 treatment was significantly higher than under H1 treatment. Under the same humidity conditions, except for 20 d when L1, L2 treatment and 40 d when H1 treatment showed no significant effect on branch length under L1 treatment, different light treatments had significant effects on branch length, and L1 < L2 < L3.

**Figure 3:**
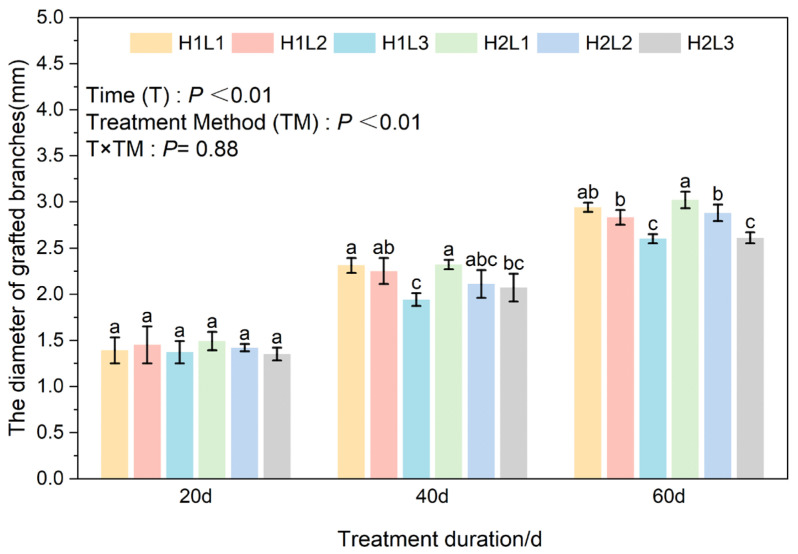
Effects of different light and humidity combinations on branch diameter of grafted seedlings of *C. betulus*. Different lowercase letters above the bars indicate significant differences between treatments (*P* < 0.05) according to Duncan’s multiple range test. Treatments sharing the same letter are not significantly different. Treatments sharing the same letter are not significantly different (e.g., ‘a’ is significantly different from ‘b’, but ‘ab’ is not significantly different from either ‘a’ or ‘b’).

In terms of branch thickness (Figure 3), under the same light conditions, the humidification treatment had no significant effect on the growth of branch diameter. At 20 d, there was no significant difference in branch diameter among the treatments. At 40 d, there was no significant difference in branch diameter between the H2L2 treatment and the other treatments. Under the same humidity conditions, the branch diameter of the L1 treatment was significantly higher than that of L3, while there was no significant difference between L2 and L1 treatments. At 60 d, under the same humidity conditions, different light treatments had a significant effect on branch diameter, and L1 > L2 > L3.

### 2.3. Effects of Humidity and Light Treatment on Number of Unfolded Leaves, Leaf Length and Width of Grafted Seedlings of C. betulus

**Figure 4:**
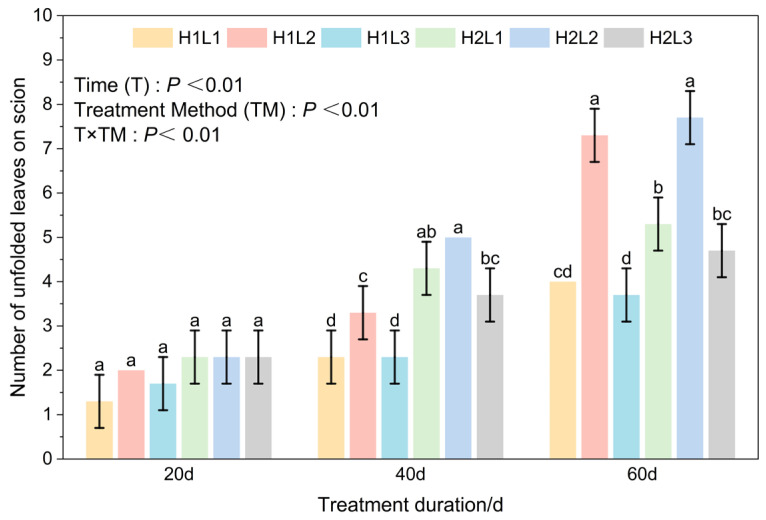
Effects of humidity and light on the number of leaves spread of grafted *C. betulus* seedlings. Different lowercase letters above the bars indicate significant differences between treatments (*P* < 0.05) according to Duncan’s multiple range test. Treatments sharing the same letter are not significantly different. Treatments sharing the same letter are not significantly different (e.g., ‘a’ is significantly different from ‘b’, but ‘ab’ is not significantly different from either ‘a’ or ‘b’).

Two-way ANOVA results showed that treatment duration, different treatments, and their interaction significantly affected the number of unfolded leaves in *C. betulus* grafted seedlings (*p* < 0.01) (Figure 4). At 20 days, there was no significant difference in the number of unfolded leaves among the treatments. At 40 days, the H2L2 treatment had the highest number of unfolded leaves, reaching 5 leaves; under the same light conditions, the number of unfolded leaves in H2-treated grafted seedlings was significantly higher than in H1 treatment; under H1 conditions, the H1L2 treatment had significantly more unfolded leaves than H1L1 and H1L3, and there was no significant difference between H1L1 and H1L3 treatments. Under H2 conditions, there were significant differences in the number of unfolded leaves between H2L1 and both H2L2 and H2L3 treatments, and there was also a clear difference between H2L2 and H2L3 treatments. At 60 days, under L1 and L3 conditions, the number of unfolded leaves in H1-treated grafted seedlings was significantly lower than in H2 treatment. Under L2 conditions, the number of unfolded leaves was significantly the highest, reaching 7.7 and 7.3 leaves, respectively, but humidity had no significant effect on the number of unfolded leaves. Under the same humidity conditions, the L2 treatment had significantly more unfolded leaves than L1 and L3 treatments, while there was no significant difference between L1 and L3.

**Figure 5:**
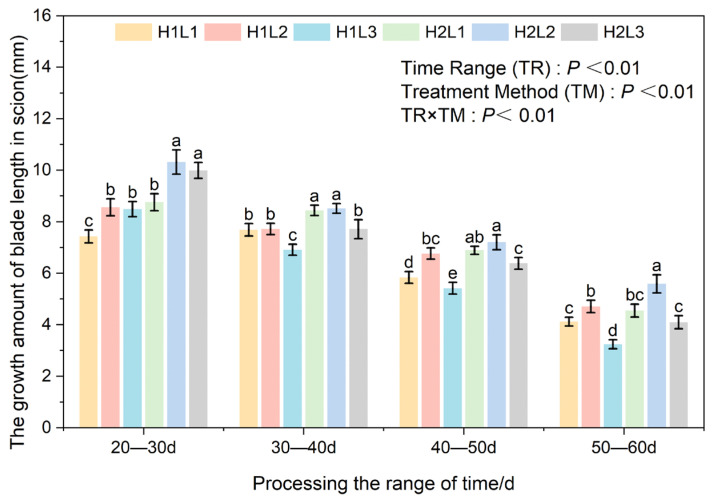
Effects of different combinations of light and humidity on leaf length of grafted seedlings of *C. betulus*. Different lowercase letters above the bars indicate significant differences between treatments (*P* < 0.05) according to Duncan’s multiple range test. Treatments sharing the same letter are not significantly different. Treatments sharing the same letter are not significantly different (e.g., ‘a’ is significantly different from ‘b’, but ‘ab’ is not significantly different from either ‘a’ or ‘b’).

Two-way ANOVA confirmed that the measurement period, environmental treatment (humidity × light), and their interaction significantly influenced the leaf morphogenesis (length and width) of *C. betulus* grafted seedlings (*p* < 0.01) (Figure 5). Generally, the leaf elongation rate exhibited a downward trend across all treatments as the leaves approached maturity, with the exception of the H1L1 treatment, which showed a fluctuation.

Across all developmental stages (20–60 d), the humidified treatment (H2) consistently resulted in significantly greater leaf length growth compared to natural humidity (H1). The effect of light intensity varied by developmental stage. In the early stage (20–30 d), moderate (L2) and low light (L3) promoted elongation better than full light (L1). However, as leaf development progressed (30–60 d), the interaction between humidity and light became prominent. The H2L2 regime (Humid + 60–70% Light) demonstrated a clear synergistic advantage, maintaining the highest growth rate throughout the middle and late stages. By the final stage (50–60 d), leaf elongation in H2L2 reached 4.09 mm, significantly outperforming all other treatments. Conversely, the combination of natural humidity and low light (H1L3) severely inhibited leaf expansion, yielding the lowest growth rates throughout most of the experiment (except for the initial 20–30 d period).

**Figure 6:**
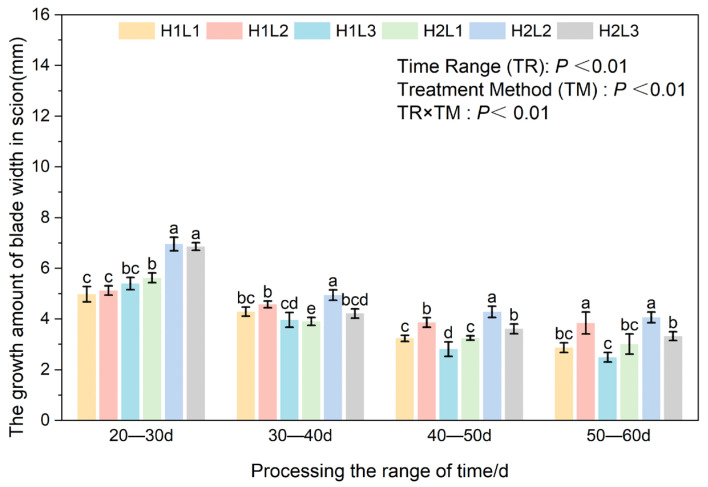
Effects of different combinations of light and humidity on leaf width of grafted seedlings of *C. betulus*. Different lowercase letters above the bars indicate significant differences between treatments (*P* < 0.05) according to Duncan’s multiple range test. Treatments sharing the same letter are not significantly different. Treatments sharing the same letter are not significantly different (e.g., ‘a’ is significantly different from ‘b’, but ‘bc’ is not significantly different from either ‘b’ or ‘c’).

Similar to leaf length, leaf width showed the characteristic of rapid growth in the early stages of all processing periods, followed by a gradual slowdown (Figure 6). Humidity played a dominant role in the initial expansion phase. At 20–30 days, the humidified treatment (H2) significantly promoted leaf width compared to natural humidity (H1) under identical light conditions. Specifically, under high humidity, shading (L2 and L3) proved more beneficial than full light (L1), suggesting that young leaves are sensitive to high irradiance. By 30–40 days, a complex interaction emerged; while H1 performed better under full light (L1), the H2L2 combination began to establish its superiority, significantly outperforming other groups under humidified conditions.

At 40–60 days, as leaves matured, the specific effect of light intensity became the determining factor. The moderate light treatment (L2) consistently resulted in the widest leaves regardless of humidity levels. At 40–50 days, L2 treatments (H1L2 and H2L2) significantly outperformed L1 and L3 counterparts. By the final stage (50–60 days), while the promoting effect of humidity plateaued in the L1 and L2 groups, the L2 light intensity maintained a significant advantage over L1 and L3.

### 2.4. Effects of Grafting Method and Period on Graft Survival

**Figure 7:**
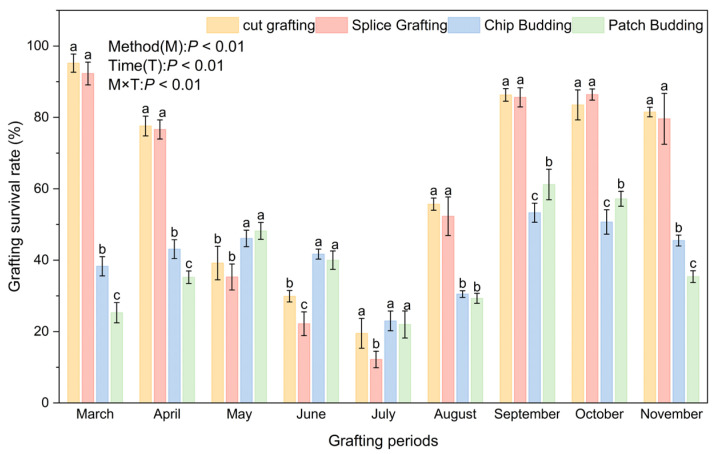
The influence of grafting methods at different periods on the survival rate of *C. betulus* grafted. Note: M = grafting method; T = grafting period; M × T = interaction of method and period. Different lowercase letters above the bars indicate significant differences between treatments (*P* < 0.05) according to Duncan’s multiple range test. Treatments sharing the same letter are not significantly different. Treatments sharing the same letter are not significantly different (e.g., ‘a’ is significantly different from ‘b’).

In order to explore the influence of different grafting methods and periods on the survival rate of grafted seedlings, we evaluated the survival rates of the four grafting methods from March to November (Figure 7). Two-way ANOVA indicated that grafting method (M), grafting period (T), and their interaction (M × T) all had significant effects on the survival rate of grafted seedlings (*p* < 0.01). The survival rates of cleft graft and whip-and-tongue graft gradually decreased from March to July, then increased rapidly and formed a secondary peak in September–October, but overall did not exceed the level observed in March. In contrast, the survival rates of grafted seedlings by chip budding and patch budding showed a trend of first increasing and then decreasing from March to July and from August to November.

During March-April and August-November, the survival rates of cleft graft and whip-and-tongue graft were significantly higher than those of chip and patch budding grafts. Among them, the survival rates of cleft graft and whip-and-tongue graft in March reached 95.2% and 92.3%, respectively, which were the highest throughout the year. The survival rates of chip budding and patch budding in May and June were significantly higher than those of cleft graft and whip-and-tongue graft. The survival rates of chip budding and patch budding in May were 46.1% and 48.2%, respectively. The survival rate of chip budding in March-April and November was significantly higher than that of patch budding. The survival rate of patch budding in September and October was significantly higher than that of chip budding. During the entire grafting period, there was no significant difference in the survival rate of grafted seedlings between the two grafting methods of cleft graft and whip-and-tongue graft.

### 2.5. Correlation Analysis of Environmental Factors and Growth Indicators

**Figure 8:**
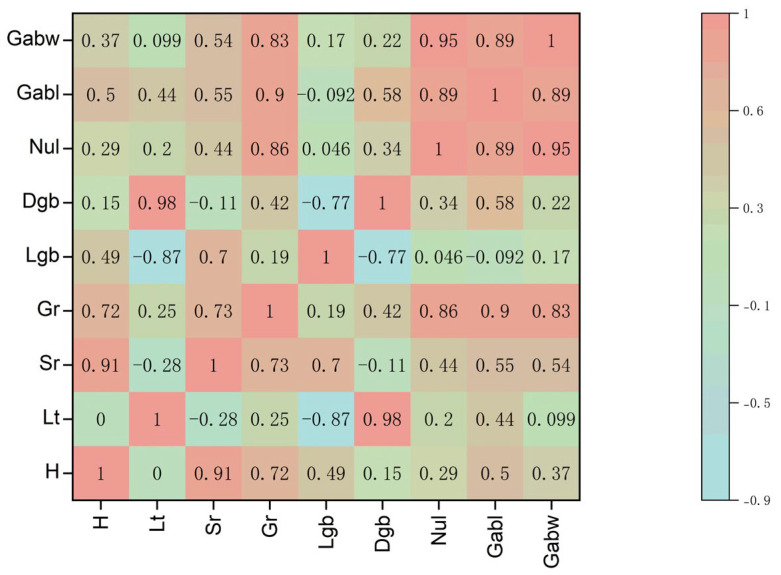
Pearson correlation heatmap between environmental factors and physiological growth indicators of *C. betulus* grafted. The color scale represents the correlation coefficient (r), ranging from −0.9 (blue, negative correlation) to 1 (pink, positive correlation). Abbreviations: H: Humidity; Lt: Light transmission; Sr: Survival rate; Gr: Germination rate; Lgb: Length of grafted branch; Dgb: Diameter of grafted branch; Nul: Number of unfolded leaves; Gabl: Growth amount of blade length; Gabw: Growth amount of blade width. (Note: Numbers inside the squares indicate the Pearson correlation coefficient values).

To systematically quantify the relationship between microclimatic treatments and seedling development, a Pearson correlation analysis was performed (Figure 8), revealing distinct regulatory strategies for humidity and light. Humidity emerged as the decisive factor for survival, exhibiting an extremely strong positive correlation with survival rate (r = 0.91) and germination rate (r = 0.72). This confirms that maintaining high hydraulic status is the prerequisite for graft survival. In contrast, light transmittance functioned as the primary regulator of morphological quality, showing a robust positive correlation with diameter of grafted branches (r = 0.98) but a strong negative correlation with length of grafted branches (r = −0.87). This divergent response quantitatively verifies the etiolation effect: under low light, seedlings prioritize vertical elongation (shade avoidance) at the expense of radial thickening, whereas sufficient light promotes stout, robust growth. Furthermore, germination rate showed a high positive correlation with growth amount of blade width (r = 0.83), indicating that early physiological vigor lays the foundation for subsequent biomass accumulation.

## 3. Discussion

### 3.1. The Effects of Grafting Methods and Periods on the Survival Rate of Grafted C. betulus

Grafting method and period are important determinants of grafting survival rate [25], and our study confirmed that graft compatibility in *C. betulus* was strictly delineated by seasonal cambial phenology. Our results showed that branch grafting (cleft/whip-and-tongue) performed in early spring (March–April) and autumn (August–November) achieved significantly higher survival rates, consistent with the optimal timing for cambial reactivation and the active phase of secondary growth. Physiologically, the success of branch grafting in spring is driven by the basipetal mobilization of auxin from swelling buds, which triggers the dedifferentiation of parenchyma cells into a callus bridge before high transpirational demands occur [26]. This supports previous findings that under favorable spring/autumn conditions, scions used for branch grafts contain more abundant nutrient reserves than those used for budding [27].

Furthermore, the survival rates of cleft graft were slightly higher than those of whip-and-tongue graft during these periods. This is likely because the contact area in cleft grafting is larger [28], spanning the phloem, cambium, and xylem, which facilitates more abundant callus formation and nutrient transport [29]. Similarly, Bausher et al. found that expanding the contact area positively correlates with survival [30]. Thus, aligning the grafting operation with the endogenous rhythms of cambial activity and maximizing vascular contact are the primary determinants of success.

Conversely, the precipitous decline in survival observed during mid-summer (July) was likely attributable to thermal inhibition rather than procedural error. In this study, July was characterized by prolonged periods of high temperature and low humidity, which constituted the primary limiting factors [27]. Temperatures exceeding 30 °C are known to induce the accumulation of reactive oxygen species at the graft interface, causing oxidative damage to the regenerating tissues [31].

Mechanistically, while bud grafting (chip and patch) demonstrated distinct advantages in early summer (May–June) due to active cambial division facilitating bark slipping [32], the thermal barrier in July negated this benefit. Prolonged high temperatures may cause cambial contraction and intensify tissue dehydration, thereby reducing bark separability and inhibiting wound healing. Notably, chip budding (containing xylem) outperformed patch budding in spring, whereas patch budding proved superior in early autumn. This indicates that when the bark is not readily separable, retaining the xylem provides essential hydraulic support [33]; conversely, during the active growth phase, the larger contact area of patch budding promotes superior healing [34]. Consequently, avoiding high-temperature periods is critical to preventing oxidative damage and ensuring the vascular cambium retains its regenerative potential.

### 3.2. The Effects of Humidity and Light on the Growth of C. betulus

Light and humidity are crucial environmental factors that have clear regulatory effects on plant developmental processes [35]. Our results revealed a significant interaction between them, confirming that graft healing requires a critical balance between moisture regulation and carbon assimilation. High humidity consistently improved survival and branch elongation. Mechanistically, humidification reduces the vapor pressure deficit surrounding the scion, thereby minimizing transpirational water loss [36]. This preservation of water potential maintains cellular turgor pressure, which is the biophysical prerequisite for cell expansion and division [37].

This accounts for the significant increase in branch elongation rates under high humidity, as the alleviation of water deficit stimulated meristematic activity. Our findings are consistent with Zhai et al., who reported that humidity enhances the regulatory efficacy of light [38]. Under water-deficit conditions (natural humidity), hydraulic disconnection limits the scion’s capacity to maintain the turgor pressure essential for vigorous callus proliferation [39]. Consequently, survival rates were significantly suppressed across all light intensities compared to humidified treatments, indicating that light regulation alone cannot compensate for a lack of hydraulic continuity. This confirms that maintaining a favorable water status is a prerequisite for successful *C. betulus* grafting [40].

Notably, the H2L2 treatment (humidification combined with 60–70% light transmission) exhibited a synergistic effect, optimizing both survival rates and leaf morphogenesis. While light signals are indispensable for callus induction [41,42] and vascular differentiation, excessive irradiance under water-deficit conditions triggers photo-oxidative stress, a condition in which unutilized light energy damages photosynthetic reaction centers [40]. We propose that high humidity enhances the scion’s physiological capacity to utilize light. By mitigating water stress, the scion is able to tolerate moderate light intensity, channeling energy into photosynthesis and vascular regeneration rather than succumbing to stress-induced necrosis.

This accounts for why the number of unfolded leaves, along with leaf length and width, reached optimal levels under 60–70% light transmission. Consistent with leaf development studies, light signals stimulate juvenile tissues to regulate leaf primordia differentiation via hormonal modulation [43]. However, this regulation is contingent upon hydraulic status. Under 20–30% light transmission, although branches elongated due to an etiolation response, insufficient photosynthetic energy limited biomass accumulation. Consequently, the H2L2 regime represents the optimal physiological equilibrium, where a minimized vapor pressure deficit facilitates maximal light utilization efficiency for leaf differentiation and expansion.

## 4. Materials and Methods

### 4.1. Test Site and Materials

During a growing season (March to November), 2-year-old *C. turczaninowii* seeds were selected and sown in a mixed substrate (vermiculite:peat soil = 1:1) in the artificial climate chamber of the Garden Experiment Teaching Center of Nanjing Forestry University. After the seedlings germinate, they should be transplanted into plastic pots (height: 20 cm, diameter: 15 cm), filled with a mixed substrate (vermiculite: peat soil: garden soil = 1:1:1), and neatly placed in the outdoor seedbed. The pots were placed directly on the soil surface, ensuring that the drainage holes at the bottom were in close contact with the field soil, which can reduce the loss of substrate water in the pot during drought. After the seedlings have grown for one year, they can be used as grafting rootstocks. At this time, the height of the rootstock seedlings was 0.7 to 0.9 m and the ground diameter was 0.7 to 0.9 cm. Cut the perennial *C. betulus* trees from a distance of 20 cm from the base to prepare scions. The roots of the basal portion will sprout many branches, so the branches should be plump and thick, with a thickness not exceeding the diameter of the part where the rootstock is grafted. In addition, the branches should carry a large number of unsprouted buds. Collect them before grafting and place them in pure water for later use.

### 4.2. Experimental Design

**Figure 9:**
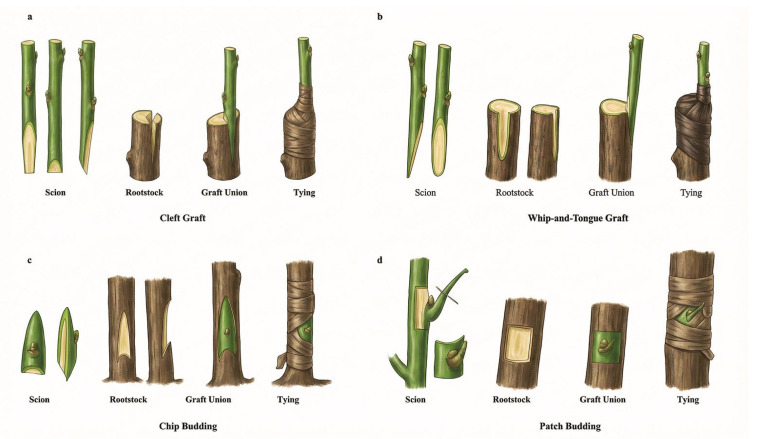
Operation flowchart of different grafting methods. (**a**) cleft graft; (**b**) whip-and-tongue graft; (**c**) chip budding; (**d**) patch budding.

The experiment adopted a completely random design. Among the grafting methods, four treatments were set, namely the cleft graft method (Figure 9a), the whip-and-tongue graft method (Figure 9b), the chip budding method (Figure 9c), and the patch budding method (Figure 9d) (Table 1). During the grafting period, nine treatments were set up, that is, grafting was carried out every month from March to November. For each treatment combination, graft 30 to 40 plants and repeat this process three times.

In the treatment related to humidity and light, the cleft graft method was uniformly adopted for grafting. During the operation, the seedlings of the rootstock were cut dry at a height of 12 cm, and then cut straight downward at an appropriate position at the upper end of the rootstock. The width of the cut was basically the same as the thickness of the scion, and the depth was about 3 cm. The scions were selected from the prepared branches of *C. betulus*, and were cut into scions with 2 buds according to the position of the buds. Cut a 3 cm bevel on the front of the lower end and a 1 cm bevel on the back end. The bottom end should be in a “—” shape. After joining, tightly bind the joining part with white plastic film. At the same time, to prevent the scion from losing water, wrap the top of the scion with plastic film as well.

Set up 6 processing combinations, that is, H1L1 (natural humidity + full light), H1L2 (natural humidity + light transmittance 60–70%), H1L3 (natural humidity + light transmittance 20–30%), H2L1 (humidification + full light), H2L2 (humidification + light transmittance 60–70%), H2L3 (humidification + light transmittance 20–30%). Using H1L1 as the CK, 30 to 40 plants were grafted in each treatment combination, and this was repeated three times to ensure the reliability of the experimental results. “Natural humidity” is usually between 30% and 80%. “Humidification” refers to the practice of spraying water on grafted seedlings at 10:00 and 15:00 every day after grafting to increase the air humidity around the grafted seedlings. The spray should form a mist of water droplets in the air, thoroughly soaking the entire seedling. The humidity was usually above 80%. The “different light transmittance” was achieved by combining the TES-1334 illuminance meter and setting up different layers of shading nets above the seedlings to change the light intensity.

### 4.3. Index Determination

Grafting survival rate: In different grafting methods and period treatment experiments, the number of surviving grafted seedlings was investigated 25 to 30 days after each grafting (for branch grafting, the survival standard was based on the fresh green color of the scion or the emergence of leaves from the buds; for bud grafting, the survival standard was based on the fresh green color of the scion skin or the emergence of leaves from the buds), and then the grafting survival rate under each treatment was calculated.

Grafting survival rate and germination rate: In experiments under different light and humidity conditions, the number of surviving plants and the number of sprouting plants in each treatment were investigated on the 20th day after grafting (fresh green scions or sprouting leaves from buds indicated that the grafting had been successful), and then the grafting survival rate and germination rate were calculated.

The length, thickness and number of spreading leaves of new branches: In experiments under different light and humidity conditions, the length, thickness and number of spreading leaves of new branches of each treated standard plant were measured, respectively, on the 20th, 40th and 60th days after grafting. The length of the new branches is measured with a ruler, and the thickness is measured with a vernier caliper.

Leaf length and width: In experiments under different light and humidity conditions, the leaf length and width of each treated standard plant were measured on the 20th, 30th, 40th, 50th, and 60th days after grafting. Then, the growth of leaf length and leaf width on the 20th to 30th days, 30th to 40th days, 40th to 50th days, and 50th to 60th days were calculated. To ensure the consistency of the measured objects, the leaves should be in the same direction and at the same position on the standard plant. When measuring leaf length and width, the leaf was laid flat on a plastic foam board, two marker pins were inserted at the two endpoints to be measured on the leaf, and the distance between the pins was then measured with a vernier caliper to indirectly obtain the leaf length or width.

### 4.4. Data Analysis

Data collation and basic calculations were performed in Microsoft Excel 2010. Statistical analyses were carried out using SPSS 18.0. Graphs were plotted with Origin 2024b. In terms of data analysis methods, one-way analysis of variance (ANOVA) was used to evaluate the effects of different treatments on the survival rate, germination rate, and growth of branches and leaves of grafted seedlings. Two-way ANOVA was utilized to analyze the interaction effects of grafting methods, grafting period, humidity and light on these indicators. Significance analysis was conducted using Duncan’s analysis, and the significance level was set at *p* < 0.05.

## 5. Conclusions

This study established a definitive correlation between the timing of grafting and the seasonal dynamics of vascular cambium activity in *C. betulus*. We concluded that grafting success was governed by the synchronization of propagation techniques with the plant’s physiological rhythms and microclimatic requirements. Notably, the survival of *C. betulus* grafts exhibited a bimodal pattern corresponding to specific physiological phases. Branch grafting (cleft/whip-and-tongue) performed in early spring (March–April) and autumn (August–November) achieved the highest survival rates, which aligned with the periods of optimal cambial reactivation and secondary thickening.

In contrast, bud grafting (chip/patch) demonstrated superior performance during early summer (May–June), a period characterized by vigorous cambial division and high cellular turgor that facilitated bark slipping. However, during midsummer (July), neither method was conducive to survival. This failure was attributed to thermal inhibition, where high temperatures (>30 °C) induced metabolic stress and dehydration, limiting the regenerative capacity of the graft interface. Therefore, practical production should prioritize branch grafting in spring/autumn and use budding in early summer as a supplemental technique to maximize annual yield.

We identified a critical synergistic interaction between humidity and light. The H2L2 regime (Humidification +60–70% Light Transmission) constituted the optimal technical combination. Under identical humidity conditions, moderate light (60–70%) significantly outperformed low light (20–30%) and full light treatments in terms of survival and leaf development. Physiologically, high humidity significantly promoted the elongation of new shoots by alleviating water deficit (especially under low light conditions), while moderate light provided the necessary photosynthetic products for vascular differentiation.

This study systematically elucidated the optimal timing, techniques, and environmental thresholds required for the large-scale propagation of *C. betulus*, providing a technical foundation for its introduction and domestication. Future research should expand beyond above-ground microclimates to explore the influence of soil physicochemical properties and rhizosphere microbial communities on graft healing. Specifically, investigating how root-zone temperature and microbial interactions regulate callus formation will further clarify the mechanisms underlying the establishment and acclimatization of grafted *C. betulus* seedlings.

## Figures and Tables

**Table 1:** Comparison of four grafting methods for *C. betulus* seedlings.

Grafting Method	Grafting Height (cm)	Incision/Interface Length (cm)	Scion/Bud Characteristics	Figure Ref.
Cleft Graft	10	2.5–3.5	Scion with 2–3 buds; Basal wedge shape.	Figure 9a
Whip-and-Tongue Graft	10	3.0–4.0	Scion with 2–3 buds; Long matching bevel cut.	Figure 9b
Chip Budding	5	~2.0	The scion has a bud; The size of the scion matches the cut of the rootstock	Figure 9c
Patch Budding	5	Matches bud size	Rectangular patch without xylem (bark only).	Figure 9d

## Data Availability

The data are not publicly available due to privacy restrictions.
